# Prognostic value of fast semi-automated left atrial long-axis strain analysis in hypertrophic cardiomyopathy

**DOI:** 10.1186/s12968-021-00735-2

**Published:** 2021-03-25

**Authors:** Fuyao Yang, Lili Wang, Jie Wang, Lutong Pu, Yuanwei Xu, Weihao Li, Ke Wan, Dan Yang, Jiayu Sun, Yuchi Han, Yanjie Zhu, Yucheng Chen

**Affiliations:** 1 Cardiology Division, Department of Medicine West China Hospital, Sichuan University, Guoxue Xiang No. 37, Chengdu, 610041 Sichuan China; 2 Department of Geriatrics, West China Hospital, Sichuan University, Sichuan Province, Chengdu, China; 3 Department of Radiology, West China Hospital, Sichuan University, Sichuan Province, Chengdu, 610041 People’s Republic of China; 4 Center of Rare diseases, West China Hospital, Sichuan University, Chengdu, 610041 China; 5 Department of Medicine (Cardiovascular Division), University of Pennsylvania, Philadelphia, PA USA; 6 Paul C. Lauterbur Research Centre for Biomedical Imaging, Shenzhen Institutes of Advanced Technology, Chinese Academy of Sciences, Guangdong, 518055 China

**Keywords:** Cardiovascular magnetic resonance, Hypertrophic cardiomyopathy, Left atrial, Strain, Prognosis

## Abstract

**Background:**

The prognostic value of left atrial (LA) size and function in hypertrophic cardiomyopathy (HCM) is well recognized, but LA function is difficult to routinely analyze. Fast LA long-axis strain (LA-LAS) analysis is a novel technique to assess LA function on cine cardiovascular magnetic resonance (CMR). We aimed to assess the association between fast LA-LAS and adverse clinical outcomes in patients with HCM.

**Methods:**

359 HCM patients and 100 healthy controls underwent routine CMR imaging. Fast LA-LAS was analyzed by automatically tracking the length between the midpoint of posterior LA wall and the left atrioventricular junction based on standard 2- and 4-chamber balanced steady-state free precession cine-CMR. Three strain parameters including reservoir strain (εs), conduit strain (εe), and active strain (εa) were assessed. The endpoint was set as composite adverse events including cardiovascular death, resuscitated cardiac arrest, sudden cardiac death aborted by appropriate implantable cardioverter-defibrillator discharge, and hospital admission related to heart failure.

**Results:**

During an average follow-up of 40.9 months, 59 patients (19.7%) reached endpoints. LA strains were correlated with LA diameter, LA volume index (LAVI) and LA empty fraction (LAEF) (all p < 0.05). In the stepwise multivariate Cox regression analysis, εs and εe (hazard ratio, 0.94 and 0.89; p = 0.019 and 0.006, respectively) emerged as independent predictors of the composite adverse events. Fast LA εs and LA εe are stronger prognostic factors than LA size, LAVI and the presence of left ventricular late gadolinium enhancement.

**Conclusions:**

Fast LA reservoir and conduit strains are independently associated with adverse outcomes in HCM.

## Background

Hypertrophic cardiomyopathy (HCM), characterized by unexplained increase in left ventricular (LV) wall thickness, is a common inherited cardiac disorder, with a prevalence of approximately 0.2% in the general population [1–3]. A recent retrospective cohort study involving 4893 adult HCM patients from seven European referral centers reported that during a median follow-up of 6.2 years, 14.7% patients reached the composite end point of all-cause mortality, heart transplant, and aborted sudden cardiac death (SCD), representing an excess mortality and morbidity compared to the general population [4]. However, identification of high risk HCM patients remains challenging.

The complex pathophysiology of HCM include LV outflow tract (LVOT) obstruction, LV diastolic dysfunction, mitral regurgitation, and myocardial ischemia [2, 5]. Left atrial (LA) enlargement and elevated LA pressure are consequences of these pathophysiologic processes and reduced LA compliance may be due to intrinsic atrial myopathy [6–8]. Several studies have reported that LA size is significantly associated with adverse clinical outcomes in patients with HCM; particularly relevant to the identification of patients at a risk of heart failure (HF)-related death [1, 9]. LA function could more better reflect LA pathophysiology and providing additional prognostic value in HCM [8].

Yang et al.found LA reservoir and conduit dysfunction before LA enlargement in patients with non-obstructive HCM by cardiovascular magnetic resonance (CMR) feature tracking (FT) [10]. More importantly, Hinojar et al.conducted a CMR-FT study and demonstrated that LA strain was associated adverse outcome [11]. But the CMR-FT algorithm automatically tracks 48 points of the LA based on various anatomic elements which is challenging to use with the complex LA anatomy, including the pulmonary veins and LA appendages [12–14].

To overcome this challenge, Leng et al.proposed a fast LA long-axis strain (LA-LAS) method for quantifying long LA deformation using routine two- and four-chamber cine-CMR image. Fast LA-LAS is less affected by the complex LA anatomy as it only involves the automatic tracking of 3 points. Compared with standard CMR-FT, they showed excellent reliability, comparability and 55% reduction in assessment time [13]. More importantly, they also confirmed that LA reservoir strain (εs) and conduit strain (εe) can provide incremental prognostic value in ST-segment elevation myocardial infarction [15].

Nevertheless, the correlation between LA-LAS and HCM prognosis remains unclear. In this study, we aimed to evaluate the prognostic value of LA-LAS, measured using a fast semi-automated CMR method, in patients with HCM.

## Methods

### Study population and design

The study population included HCM patients who underwent clinical CMR imaging between August 2011 and October 2017, and 100 healthy controls. Healthy subjects were chosen from our database to match the age of the HCM population [16]. The HCM diagnostic criteria recommended by the latest European Society of Cardiology guidelines were applied: normal LV size with LV wall thickness (WT) ≥ 15 mm in ≥ 1 LV myocardial segments that was not explained by the secondary cause of hypertrophy or WT of ≥ 13 mm with a family history of HCM [1]. The obstructive LVOT was defined as an instantaneous peak Doppler LVOT pressure gradient of ≥ 30 mm Hg with provocation or at rest [1]. The exclusion criteria included known causes of myocardial hypertrophy, contraindication for CMR, and atrial fibrillation at the time of imaging. The study protocol was approved by the Institutional Ethics Committee of West China Hospital, Sichuan University. Written informed consent was obtained from all study participants.

### CMR imaging

All participants were examined using a 3 T CMR scanner (Magnetom Tim Trio; Siemens Healthineers, Erlangen, Germany) with a 32-channel phased array cardiac coil. The consecutive short-axis covering the entire LV and three long-axis (2-, 3-, and 4-chamber) cine series were acquired using balanced steady-state free precession (bSSFP) during breath-hold (field of view = 320–340 mm^2^; slice thickness = 8 mm with no gap; repetition time = 3.4 ms; echo time = 1.3 ms; flip angle = 50°; acquisition matrix = 256 × 144; acquired temporal resolution = 42 ms; spatial resolution = 1.4 × 1.3 mm^2^). Late gadolinium enhancement (LGE)-CMR was performed using an inversion recovery turbo fast low-angle shot sequence with phase-sensitive reconstruction and acquired 10–15 min after the intravenous administration of a contrast agent (Magnevist; Bayer Schering Pharma, Berlin, Germany, 0.15 mmol/kg body weight; flip angle = 20°; matrix = 256 × 144).

### Imaging analysis

All images were analyzed using post-processing software (Medis suit 2.1, QMass 8.1, Qstrain 2.0, Medis, Leiden, the Netherlands). Biventricular volume and function, LV mass, maximal WT were assessed as previously described [16, 17]. The presence of LGE was visually assessed by two independent observers (YC and JS with more than 10 years of CMR experience) who were blinded to the clinical data. Semi-automated quantification of LGE was performed using the myocardium signal intensity of 6 standard deviations (SD) from the normal myocardium and calculated as the proportion of the LV myocardium, as described previously [16, 18].

### LA measurements

LA size was measured at the end of the LV systolic phase on 3-chamber cine images. maximal LA volume (LAV) at LV end-systole (LAV max), LA volume at LV diastole before LA contraction (LAV p-ac) and minimal LA volume at LV end-diastole (LAV min) were measured using the biplane area-length method by tracing the LA endocardial contour manually in 2- and 4-chamber cine views with the exclusion of pulmonary veins and LA appendage. Total LA empty fraction (LAEF), passive LAEF, and active LAEF were calculated using LA volumetric parameters and were consistent with those used in our previous study [19].

### Fast LA-LAS

**Fig. 1:**
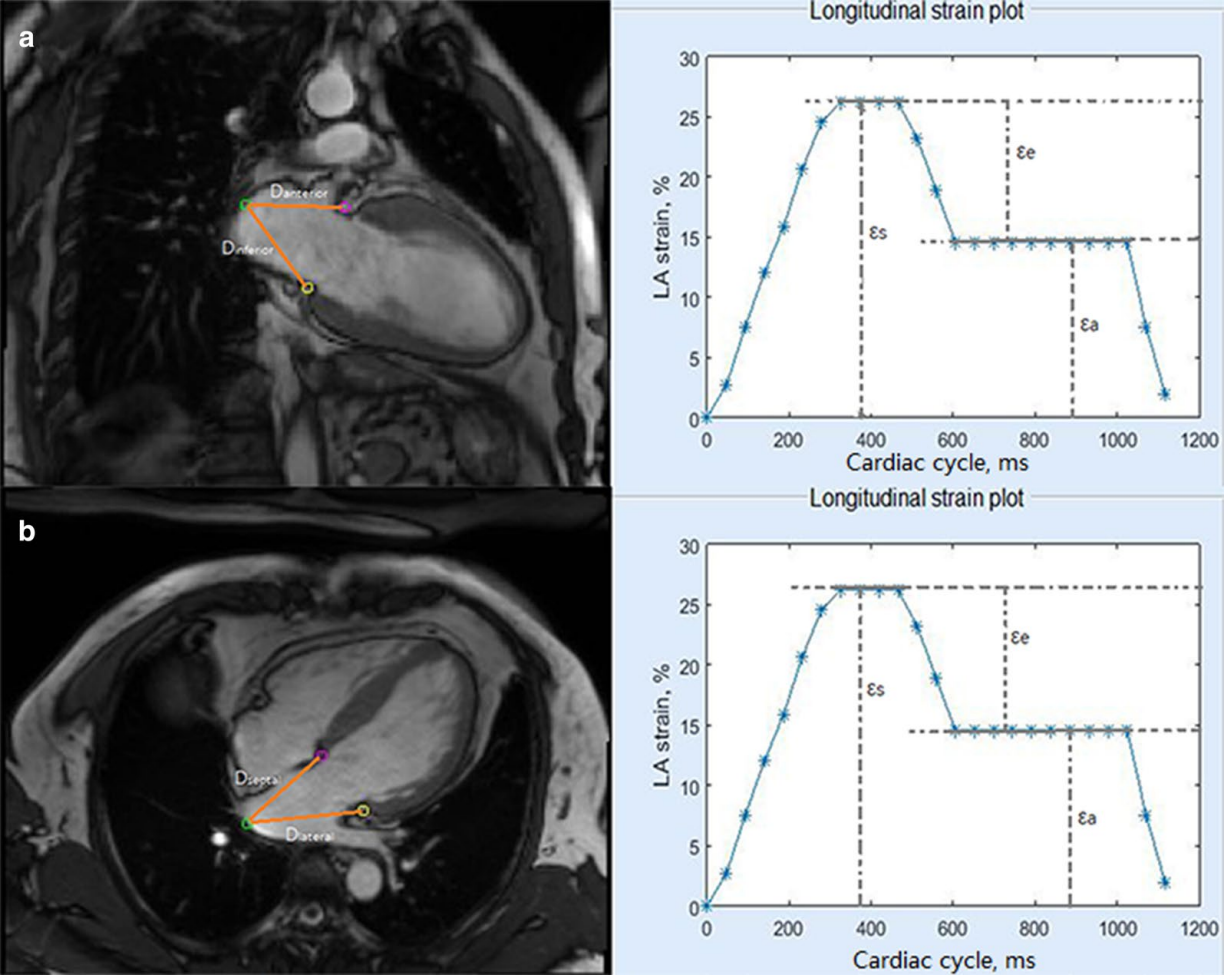
Fast left atrial (LA) long-axis strain (LA-LAS) was measured in 2- and 4-chamber views. Three small squares were manually selected at three anatomical reference points that were automatically tracked in the cardiac cycle. D: the length between the midpoint of posterior LA wall and the left atrioventricular junction on standard cine-CMR 2-chamber (D_anterior_ and D_inferior_) and 4-chamber (D_septal_ and D_lateral_) views. *ms* millisecond

LA-LAS was acquired by semi-automated tracking of the length between the midpoint of posterior LA wall and the atrioventricular junction on cine-CMR 2- and 4-chamber views (Fig. 1). Analysis was performed using a custom-written program in Matlab software (R2017a, The Mathworks, Natick, MA, USA). The midpoint of posterior LA wall was selected at the intersection point of the LA long axis and posterior LA wall. The AV junctions were defined as the mitral valve insertion points at the inferior and anterior annular insertion points on the 2-chamber view and the lateral and septal borders of the annulus on the 4-chamber view. The AV junction points and the mid-point of the posterior LA wall were manually placed at end-diastole and were then automatically tracked on other cardiac phases using the method of template matching. The automatic tracking results were manually adjusted when these points failed to track on the cine images with slight artifacts. The analysis method of fast LA-LAS was adopted from the method published and validated against CMR-FT method [13].

LA-LAS (ε) was calculated using the following formula: ε (t) = [D (t) − D_0_] × 100/D_0_ (t: time points in the cardiac cycle, 0: LV end-diastolic phase). Fast LA-LAS was assessed at three phases: εs, εe, and actrive strain (εa). Finally, mean values of LA strain in 2- and 4-chamber views were used for further analyses.

### Reproducibility

To examine variability, a sample of 30 patients and 30 healthy controls were randomly selected for reproducibility assessment. For intra-observer variability, the same investigator analyzed LA-LAS of the selected sample at least two weeks later blinded to the first analysis results. For inter-observer variability, a different investigator who were blinded to the clinical data and results by the first observer reanalyzed the data.

### Clinical follow-up

The follow-up period was the duration between the initial clinical evaluation, which was performed at the first CMR, and October 2019. Follow-up information was obtained from direct communication with patients or their family members. The combined outcome was defined as a composite of cardiovascular death, resuscitated cardiac arrest, SCD aborted by appropriate implantable cardioverter-defibrillator discharge (ICD), and hospital admission related to HF.

### Statistical analysis

Categorical variables are expressed as N (%); continuous variables are expressed as mean ± SD or median with interquartile range. Student’s t-test was used to compare two normally distributed variables. For comparing the proportions of categorical variables, chi-square test and Fisher’s exact test were applied, as appropriate. The correlation between fast LA strains and other parameters was assessed using Pearson’s and Spearman’s correlation coefficients, as appropriate. Receiver operating curve analysis was used to determine optimal cut-off values. Survival curves were established using the Kaplan–Meier method and compared using the log-rank test. Hazard ratio (HR) and corresponding 95% confidence interval (CI) were calculated by the univariate Cox proportional hazards regression analysis. Variables with p < 0.1 in the univariate Cox regression analysis were included in the stepwise multivariate Cox regression analysis to identify independent factors. Inter- and intraobserver reproducibility of fast LA-LAS was evaluated by calculating the coefficient of variation (COV) and intraclass correlation coefficient (ICC). Two-tailed p < 0.05 indicated statistical significance. Statistical analysis was performed using MedCalc (version13.0, Ostend, Belgium) and SPSS (version17.0, Statistical Package for the Social Sciences, International Business Machines, Inc., Armonk, New York, USA).

## Results

### Baseline clinical characteristics

A total of 386 HCM patients were enrolled. After excluding 27 patients for poor cine images or arrhythmia, 359 patients with HCM and 100 healthy controls were included in the analysis. Baseline clinical and CMR data of patients with HCM and healthy controls are summarized in Tables 1 and 2. In comparison with healthy controls, HCM patients had larger LV volume, lower LV ejection fraction (LVEF), larger LV mass index (LVMI), lower right ventricular (RV) end-systolic volume index (RVESVI), larger LAV, and lower LAEF.

**Table 1:** Baseline characteristics of HCM patients and healthy volunteers

Variable	Healthy subjects (n = 100)	HCM (n = 359)	P	HCMR Patients without combined outcome (n = 300)	HCM Patients with combined outcome (n = 59)	P	HCM Patients with LA diameter > 41 mm (n = 135)	HCM Patients with LA diameter ≤ 41 mm (n = 224)	P
Clinical data									
Age, years	47 (36,66)	49 (39,60)	0.746	48 (36,55)	60 (49,69)	< 0.001*	52.3 ± 13.3	45.2 ± 17.3	< 0.001*
Male gender, n (%)	43 (43.0)	198 (55.2)	0.031*	171 (57.0)	27 (45.7)	0.113	64 (47.4)	134 (59.8)	0.022
BMI (kg/m^2^)	22.7 (20.7,24.3)	23.7 (21.5,25.6)	0.004*	23.7 (21.5,25.5)	23.6 (21.3,25.8)	0.754	24.2 (22.2,26.2)	23.3 (20.8,25.4)	0.001*
BSA (m^2^)	1.6 ± 0.2	1.7 ± 0.2	< 0.001*	1.7 ± 0.2	1.6 ± 0.2	0.033*	1.7 (1.6,1.8)	1.7 (1.5,1.8)	0.316
Heart rate (beat/minute)	72 (66,78)	73 (65,77)	0.654	73 (66,76)	71 (63,78)	0.431	73 (65,80)	73 (66,75)	0.164
Diabetes mellitus, n (%)	–	24 (6.7)	–	20 (6.7)	4 (6.8)	1.000	12 (8.9)	12 (5.4)	0.194
Hypertension, n (%)	–	82 (22.8)	–	62 (20.7)	20 (33.9)	0.027*	36 (26.7)	46 (20.5)	0.180
Obstructive HCM, n (%)	–	137 (38.2)	–	103 (34.3)	34 (57.6)	0.001*	67 (49.6)	70 (31.3)	0.001*
Family history of SCD, n (%)	–	49 (13.6)	–	39 (13.0)	10 (16.9)	0.419	18 (13.3)	31 (13.8)	0.892
History of syncope, n (%)	–	65 (18.1)	–	50 (16.7)	15 (25.4)	0.110	24 (17.8)	41 (18.3)	0.900
CAD, n (%)	–	27 (7.5)	–	21 (7.0)	6 (10.2)	0.566	15 (11.1)	12 (5.4)	0.045*
Cardiac medications									
β blocker, n (%)	–	239 (66.6)	–	194 (64.7)	45 (76.3)	0.084	102 (75.6)	137 (61.2)	0.005*

*BMI*  body mass index, *BSA*  body surface area, *CAD* coronary artery disease,  *HCM*  hypertrophic cardiomyopathy,  *SCD*  sudden cardiac death

*p < 0.05; Data are expressed as mean ± SD, mean with 25th–75th percentiles in parentheses, or as a percentage for categorical variables

**Table 2:** Cardiovascular magnetic resonance (CMR) left ventricular (LV) and right ventricular (RV) parameters of HCM patients and healthy subjects

Variable	Healthy subjects (n = 100)	HCM (n = 359)	P	HCM Patients without combined outcome (n = 300)	HCM Patients with combined outcome (n = 59)	P	HCM Patients with LA diameter > 41 mm (n = 135)	HCM Patients with LA diameter ≤ 41 mm (n = 224)	P
LVEF (%)	66.7 (62.3,70.4)	64.3 (58.7,68.9)	0.001*	64.6 (59.2,69.2)	62.3 (58.2,67.6)	0.044*	63.3 (56.8,69.2)	64.8 (59.4,68.7)	0.130
LVEDVI (mL/ m^2^)	71.7 (63.0,80.8)	78.5 (68.3,91.2)	< 0.001*	77.5 (68.3,87.8)	86.2 (29.2,108.8)	0.004*	85.2 (73.1,99.9)	76.3 (66.5,84.7)	< 0.001*
LVESVI (mL/ m^2^)	23.9 (19.4,29.2)	28.3 (21.9,34.9)	< 0.001*	27.5 (21.7,34.3)	32.9 (25.0,39.1)	0.002*	30.6 (23.2,38.8)	27.1 (21.3,33.9)	0.001*
RVEF (%)	60.8 (58.1,64.3)	62.0 (55.7,67.4)	0.291	61.4 ± 8.8	60.1 ± 9.2	0.260	61.2 (55.4,67.3)	62.1 (56.0,67.5)	0.274
RVEDVI (mL/ m^2^)	66.6 (57.5,78.2)	65.8 (55.0,74.0)	0.162	67.0 ± 15.3	61.8 ± 14.6	0.059	64.7 (56.0,72.3)	66.5 (55.0,75.8)	0.246
RVESVI (mL/ m^2^)	26.4 (22.4,30.9)	24.2 (19.1,30.9)	0.024*	24.4 (19.1,31.0)	24.0 (18.9,29.1)	0.538	24.2 (19.7,30.1)	24.1 (18.0,31.0)	0.775
LVMI (g/m^2^)	49.6 (43.5,54.0)	94.4 (74.9,115.4)	< 0.001*	92.2 (72.0,109.6)	99.5 (83.0,125.2)	0.006*	101.7 (82.3,124.5)	87.0 (69.3,107.3)	< 0.001*
Max WT (mm)	–	21.8 (18.1,26.0)	–	21.5 (18.0,25.8)	22.9 (20.1,26.5)	0.143	22.4 (19.1,26.0)	21.5 (17.5,26.0)	0.128
LGE extent (%)	–	5.7 (2.0,11.4)	–	5.4 (2.1,10.7)	8.3 (3.3,16.2)	0.014*	5.1 (1.6,11.8)	5.8 (2.1,11.3)	0.858

*p < 0.05; Data are expressed as mean ± SD, mean with 25th–75th percentiles in parentheses, or as a percentage for categorical variables. CMR = cardiovascular magnetic resonance; *HCM* hypertrophic cardiomyopathy, *EDVI* end-diastolic volume index, *ESVI* end-systolic volume index, *LVEF* left ventricular ejection fraction, *RVEF*  right ventricular ejection fraction, *LVMI* LV mass index, *Max WT* maximal (LV) wall thickness, *LGE* late gadolinium enhancement, *LV* left ventricular, *RV* right ventricular

### Fast LA strain measurements

The mean values of fast LA-LAS (εs, εe, and εa) in HCM patients were significantly lower than those of healthy controls (Table 3, all p < 0.01). Patients with the combined outcome showed significantly lower LA εs and LA εe than those without the combined outcome (Table 3, all p < 0.01).

**Table 3:** Left atrial parameters and LA longitudinal strain of HCM patients and healthy volunteers

Variable	Healthy subjects (n = 100)	HCM (n = 359)	P	HCM Patients without combined outcome (n = 309)	HCM Patients with combined outcome (n = 50)	P	HCM Patients with LA diameter > 41 mm (n = 135)	HCM Patients with LA diameter ≤ 41 mm (n = 224)	P
LA parameters									
LA diameter, mm	29.9 (26.6,33.4)	40.0 (34.0,45.0)	< 0.001*	39.0 (34.0,44.0)	42.0 (37.0,48.0)	< 0.001*	47.0 (44.0,50.0)	36.0 (32.0,39.0)	< 0.001*
LAVI max, ml/m2	38.0 (33.1,45.4)	59.3 (45.0,76.0)	< 0.001*	56.8 (44.1,72.8)	68.4 (54.8,89.3)	< 0.001*	75.3 (60.5,98.7)	51.4 (41.2,64.5)	< 0.001*
LAVI p-ac, ml/m2	24.4 (20.7,35.6)	45.4 (33.5,63.1)	< 0.001*	43.7 (32.1,60.4)	55.9 (43.5,78.3)	< 0.001*	64.9 (49.5,83.8)	38.2 (29.6,49.6)	< 0.001*
LAVI min, ml/m2	15.3 (11.5,18.4)	31.1 (21.1,45.3)	< 0.001*	29.3 (20.3,42.6)	39.8 (27.0,61.4)	< 0.001*	45.8 (33.1,67.9)	24.8 (18.3,35.5)	< 0.001*
Total LAEF, %	61.4 (56.2,65.8)	46.3 (37.4,54.5)	< 0.001*	46.8 ± 12.4	37.8 ± 11.5	< 0.001*	38.2 (27.0,47.0)	50.5 (42.8,56.7)	< 0.001*
Passive LAEF, %	34.4 (28.1,40.9)	20.2 (12.6,27.6)	< 0.001*	21.5 (13.3,28.3)	15.7 (10.7,21.2)	< 0.001*	13.9 (9.9,21.3)	23.1 (17.6,30.8)	< 0.001*
Active LAEF, %	41.3 (32.3,48.5)	31.9 (23.5,39.3)	< 0.001*	32.3 ± 11.3	26.2 ± 10.5	< 0.001*	26.4 ± 12.5	33.4 ± 10.9	< 0.001*
LA longitudinal strain									
Reservoir εs, %	36.1 ± 7.7	20.4 ± 7.6	< 0.001*	21.7 ± 7.4	16.6 ± 5.6	< 0.001*	15.6 (11.2,22.5)	21.9 (17.7,26.7)	< 0.001*
Conduit εe, %	20.7 (15.0,24.4)	8.8 (5.7,13.0)	< 0.001*	10.0 (6.1,13.4)	6.6 (4.9,8.1)	< 0.001*	6.6 (4.8,10.0)	10.7 (7.1,14.2)	< 0.001*
Booster εa, %	15.7 ± 4.1	11.0 ± 4.2	< 0.001*	11.3 ± 4.1	9.9 ± 4.2	0.054	9.2 (6.1,12.8)	11.4 (9.2,14.4)	< 0.001*

*p < 0.05; Abbreviations as in Table1 and Table 2. Data are expressed as mean ± SD, mean with 25th–75th percentiles in parentheses, or as a percentage for categorical variables

*LAEF *= left atrial empty fraction; *LAVI max*  maximal left atrial volume index; *LAVI p-ac* left atrial volume index prior to atrial contraction; *LAVI min*  minimal left atrial volume index; *ε*  left atrial strain

### Association among fast LA-LAS, LGE, and volumetric measurements

**Fig. 2:**
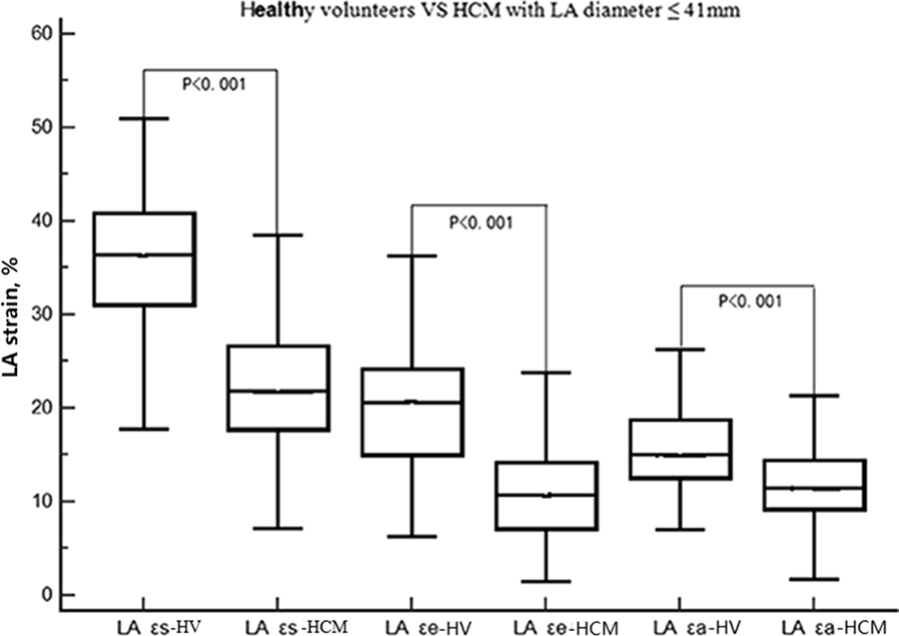
Comparison between healthy subjects with hypertrophic cardiomyopathy (HCM) patients of LA size ≤ 41 mm for the fast LA strain. *LA* left atrial, *HV* healthy subjects, *εs* reservoir strain, *εe* conduit strain, *εa* active strain

Fast LA-LAS showed strong correlation with LAEF (r: 0.79 to 0.67; Table 4, all p < 0.05), moderate correlation with LA diameter and LAV index (LAVI) (r: − 0.53 to − 0.32), and weak association with the extent of LGE ( r: − 0.17 to − 0.11). Using the upper limit for the LA anteroposterior dimension in the Chinese population of 41 mm as the LA diameter cutoff [19], the increased LA diameter group showed significantly decreased LA εs, LA εe, and LA εa (Table 3, all p < 0.01). Compared with healthy controls, fast LA-LAS (εs, εe, and εa) in patients with normal LA size (≤ 41 mm) was also significantly impaired (Fig. 2, all p < 0.01).

**Table 4:** Bivariate correlation between LGE, LA diameter, LA volumetric measurements and corresponding fast strain parameters in HCM patients

Phase	LGE/LA diameter/volumetric measurements	Correlation coefficient	P value
Reservoirεs	LGE extent	− 0.17	0.002*
	LA diameter	− 0.43	< 0.001*
	Total LAEF	0.79	< 0.001*
	LAVI max	− 0.51	< 0.001*
Conduit εe	LGE extent	− 0.11	0.048*
	LA diameter	− 0.36	< 0.001*
	Passive LAEF	0.67	< 0.001*
	LAVI p-ac	− 0.52	< 0.001*
Booster pump εa	LGE extent	− 0.15	0.005*
	LA diameter	− 0.32	< 0.001*
	Active LAEF	0.68	< 0.001*
	LAVI min	− 0.53	< 0.001*

*EF* ejection fraction, *LGE* late gadolinium enhancement, *LAEF* left atrial empty fraction, *LAVI* max maximal left atrial volume index, *LAVI p-ac* left atrial volume index prior to atrial contraction, *LAVI min* minimal left atrial volume index, *ε* left atrial strain

^*^p < 0.05

### Outcome analysis

Fifty-nine patients achieved the combined outcome during an average follow-up of 40.9 ± 19.8 months, including 11 (18.6%) cardiovascular deaths, 2 (3.4%) SCD aborted by appropriate ICD discharge, 5 (8.5%) resuscitations after syncope, and 41 (69.5%) hospital admissions related to HF. In comparison to patients without the combined outcome, those with the combined outcome were older, had more obstructive HCM, lower LVEF, lower LV end-diastolic volume index (LVEDVI), lower LV end-systolic volume index (LVESVI), lower LVMI, higher LGE extent, larger LAV, and lower LAEF (all p < 0.05).

**Fig. 3:**
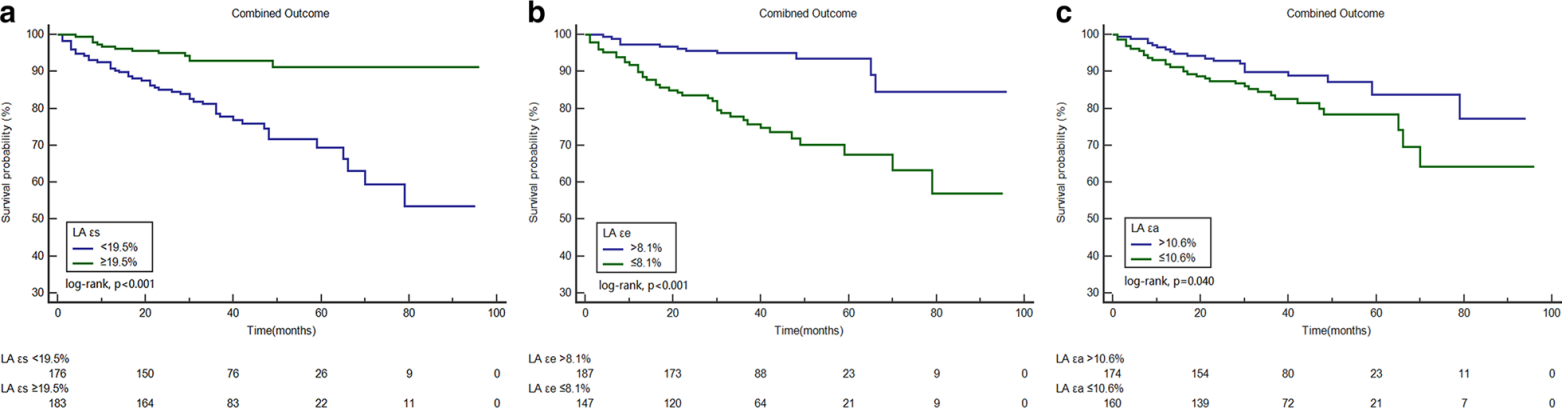
Kaplan–Meier curves demonstrating cumulative event-free survival according to left atrial longitudinal strains. **a**–**c** Grouped according to the cut-off value of εs, εe, and εa, respectively. *LA* left atrial, *εs* reservoir strain, *εe* conduit strain, *εa* active strain

**Fig. 4:**
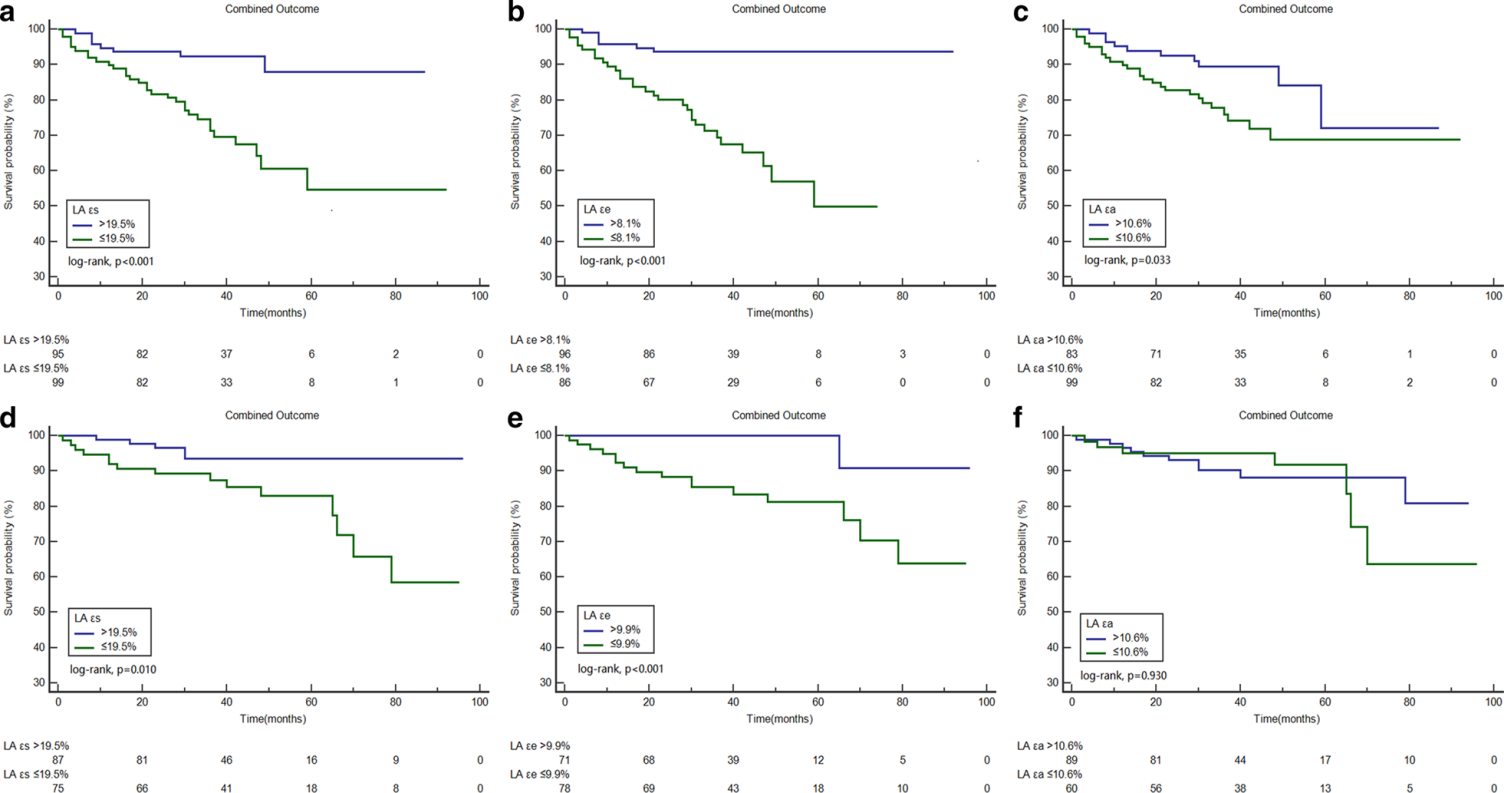
Graphs showing Kaplan–Meier cumulative survival by left atrial longitudinal strains in HCM patients with late gadolinium enhancement (LGE) (**a**–**c**) and without LGE (**d**–**f**). *LA* left atrial, *εs* reservoir strain, *εe* conduit strain, *εa* active strain

**Fig. 5:**
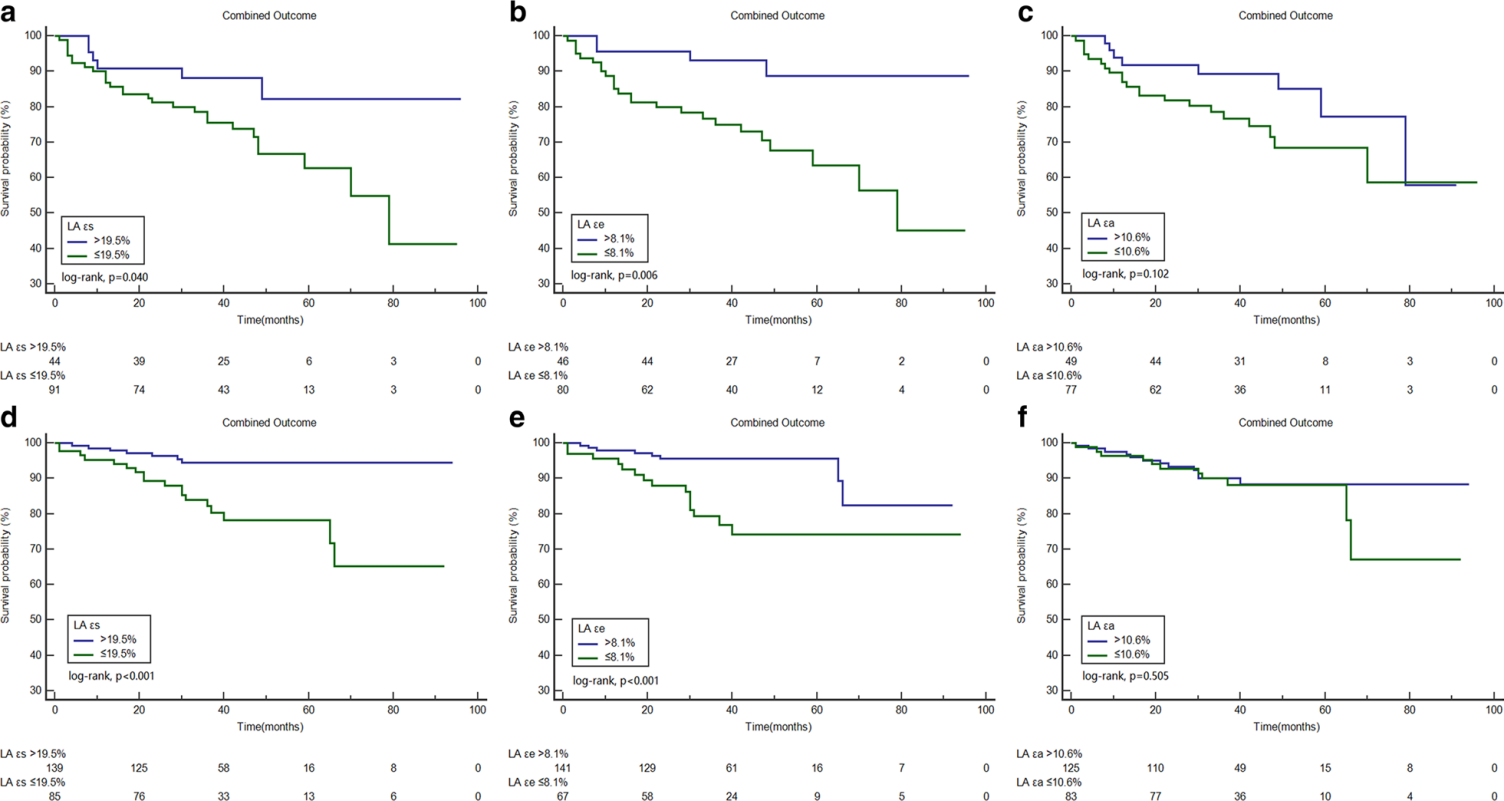
Graphs demonstrating cumulative event-free survival according to left atrial longitudinal strains in HCM patients with LA diameter > 41.0 mm (**a**–**c**) and LA diameter ≤ 41.0 mm (**d**–**f**). **a**, **d** grouped according to the cut-off value of εs; B and E, grouped according to the cut-off value of εe; and **c**, **d** grouped according to the cut-off value of εa. *LA* left atrial, *εs* reservoir strain, *εe* conduit strain, *εa* active strain

**Fig. 6:**
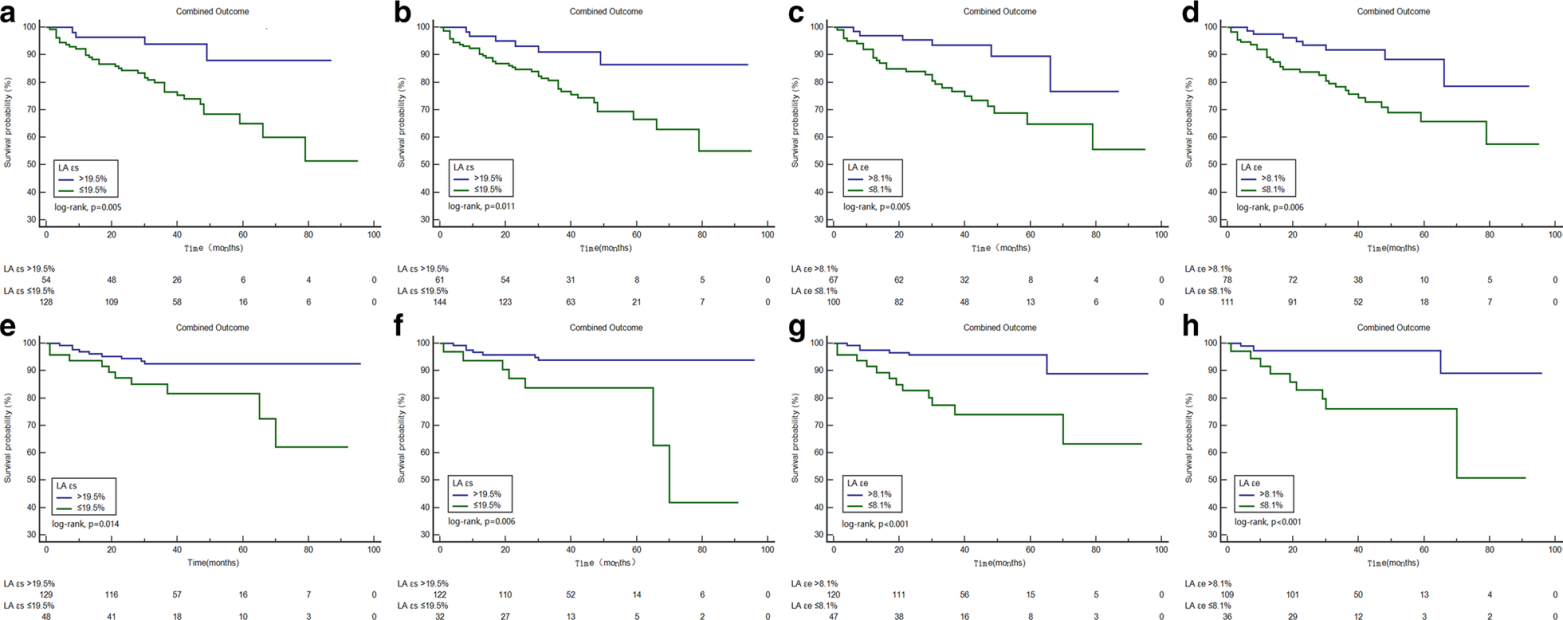
Graphs demonstrating cumulative event-free survival according to left atrial longitudinal strains in HCM patients with LAVI max > 59.1 ml/m^2^ (**a**, **c**), LAVI min > 27.8 ml/m2 (**b**, **d**), LAVI max ≤ 59.1 ml/m2 (**e**, **g**) and LAVI min ≤ 27.8 ml/m2 (**f**, **h**). **a**, **b**, **e**, **f** grouped according to the cut-off value of εs; **c**, **d**, **g**, **h** grouped according to the cut-off value of εe. *LA* left atrial, *εs* reservoir strain, *εe* conduit strain, *LAVI max* maximal left atrial volume index, *LAVI min* minimal left atrial volume index

Based on receiver operating curve analysis, the best cutoff values for fast LA εs, εe, and εa to predict the combined outcome were 19.5% (AUC = 0.70), 8.1% (AUC = 0.72), and 10.6% (AUC = 0.59), respectively (all p < 0.05). Kaplan–Meier curves for the combined outcome indicated that patients with impaired LA εs, LA εe, and LA εa achieved a significantly higher rate of the combined outcome (p < 0.05, Fig. 3). Figure 4 shows comparisons of the combined outcome among patients with impaired LA εs, LA εe, and LA εa stratified based on the presence of LGE. Impaired LA εs, LA εe, and LA εa were associated with the combined outcome in patients with positive LGE (all p < 0.05). In the subgroup of LGE negative patients, those with impaired LA εs and LA εe also had a significantly higher rate of the combined outcome (all p < 0.05), but there was no significant difference in the survival rate in those LGE negative patients with LA εa > 10.6% and those with LA εa ≤ 10.6% (p = 0.93). Figure 5 demonstrates the subgroup survival analysis based on LA diameter. Impaired LA εs and LA εe significantly increased the risk of the combined outcome irrespective of LA diameter (all p < 0.05); however, no significant differences were found between patients with impaired LA εa or preserved LA εa in this subgroup (p = 0.17). We selected the upper limit of LAVI max and LAVI min as reference (59.1 ml/m^2^, 27.8 ml/m^2^, respectively) [19]. Figure 6 demonstrated that impaired LA εs and LA εe were significantly associated with a higher risk of the combined outcome in patients with LAVI max > 59.1 ml/m^2^, and LAVI min > 27.8 ml/m^2 (^Fig. 6a–d, p < 0.05), and in patients with LAVI max ≤ 59.1 ml/m^2^ and LAVI min ≤ 27.8 ml/m^2^ (Fig. 6g, h, all p < 0.05).

### Cox regression analysis

The results of univariate Cox regression analyses are summarized in Table 5. Fast LA εs and LA εe emerged as predictors of the combined outcome. The results of stepwise multivariate proportional hazard analyses for the combined outcome are summarized in Tables 6 and 7. When fast LA-LAS was not included as a covariate, age (HR, 1.05), LVMI (HR, 1.01), LGE extent (HR, 1.03), and LAVI min (HR, 1.01) were associated with the combined outcome (Model 1, all p < 0.05). When LA εs was included as a covariable to adjust Model 1, age (HR, 1.04), LVMI (HR, 1.01), LGE extent (HR, 1.03), and LA εs (HR, 0.94) were identified to be independent predictors of the combined outcome (Model 2, all p < 0.05). When LA εe was included as a covariable to adjust Model 1, age (HR, 1.03), LVEDVi (HR, 1.01), and LA εe (HR, 0.89) were associated with the combined outcome (Model 3, all p < 0.05). When all parameters of LA-LAS were included as covariables, age, LVEDVI and εe continued to be independent predictors of the combined outcome (Model 4, all p < 0.05). However, when LA εa was included as a covariable to adjust Model 1, εa was not an independent predictor of the combined outcome in the stepwise multivariate Cox regression analysis (Table 7).

**Table 5:** Univariate analysis of all patients (n = 359) for combined outcome

Variable	Combined outcome		
	HR	95%CI	P
Age, years	1.04	1.02–1.06	< 0.001
Gender	1.48	0.89–2.47	0.133
BSA (m^2^)	0.36	0.13–0.99	0.049
BMI (kg/m^2^)	1.01	0.94–1.08	0.866
Heart rate (beat/minute)	0.99	0.97–1.02	0.529
Hypertension	1.72	1.00–2.95	0.049
Diabetes mellitus	0.98	0.36–2.71	0.972
Obstructive HCM	2.28	1.36–3.82	0.002
Family history of SCD	1.43	0.72–2.82	0.304
History of syncope	1.57	0.88–2.82	0.134
Coronary artery disease	1.53	0.66–3.57	0.322
β blocker	0.67	0.37–1.23	0.194
LVEF (%)	0.97	0.95–1.00	0.024
LVEDVI (mL/ m^2^)	1.02	1.01–1.03	< 0.001
LVESVI (mL/ m^2^)	1.02	1.01–1.03	< 0.001
RVEF (%)	0.99	0.96–1.01	0.294
RVEDVI (mL/ m^2^)	0.99	0.97–1.00	0.092
RVESVI (mL/ m^2^)	0.99	0.96–1.02	0.437
LVMI (g/m^2^)	1.01	1.00–1.02	0.001
Max WT (mm)	1.02	0.98–1.06	0.311
LGE extent (%)	1.04	1.02–1.07	0.001
LA diameter, mm	1.07	1.04–1.11	< 0.001
LAVI max, ml/m^2^	1.02	1.01–1.02	< 0.001
LAVI p-ac, ml/m^2^	1.02	1.01–1.02	< 0.001
LAVI min, ml/m^2^	1.02	1.01–1.02	< 0.001
Total LAEF, %	0.96	0.94–0.98	< 0.001
Passive LAEF, %	0.95	0.92–0.98	0.003
Active LAEF, %	0.96	0.94–0.98	< 0.001
Reservoir εs, %	0.91	0.88–0.95	< 0.001
Conduit εe, %	0.84	0.78–0.91	< 0.001
Booster εa, %	0.94	0.88–1.01	0.072

*CMR* cardiovascular magnetic resonance, *HCM* hypertrophic cardiomyopathy, *EDVI* end-diastolic volume index, *ESVI* end-systolic volume index, *EF* ejection fraction, *LVMI* LV mass index, *Max WT* maximal (LV) wall thickness, *LGE* late gadolinium enhancement, *LV* left ventricular, *RV* right ventricular, *LAEF* left atrial empty fraction, *LAVI* max maximal left atrial volume index, *LAVI* p-ac left atrial volume index prior to atrial contraction, *LAVI* min minimal left atrial volume index, *ε* left atrial strain, *BMI* body mass index, *BSA* body surface area, *EF* ejection fraction, *SCD* sudden cardiac death, *CAD* coronary artery disease

**Table 6:** Stepwise multivariate proportional hazard analyses for combined outcome

Variable	Combined Outcome							
	Model 1		Model 2		Model 3		Model 4	
	P	HR (95%CI)	P	HR (95%CI)	P	HR (95%CI)	P	HR (95%CI)
Age	< 0.001	1.05 (1.02–1.07)	< 0.001	1.04 (1.02–1.07)	0.012	1.03 (1.01–1.05)	0.012	1.03 (1.01–1.05)
LVEDVI					0.008	1.01 (1.00–1.02	0.008	1.01 (1.00–1.02)
LVMI	0.002	1.01 (1.00–1.02)	0.003	1.01 (1.00–1.02)				
LGE extent	0.015	1.03 (1.01–1.06)	0.046	1.03 (1.00–1.05)				
LAVI min	0.009	1.01 (1.00–1.02)						
Reservoir εs			0.019	0.94 (0.90–0.99)				
Conduit εe					0.006	0.89 (0.82–0.97)	0.006	0.89 (0.82–0.97)

In multivariate model 1, no left atrial longitudinal strain was included as a covariate. In multivariate model 2, reservoir εs was included as a covariate. In multivariate model 3, conduit εe was included as a covariate. In multivariate model 4, all parameters of left atrial longitudinal strain were included as covariates

*LAVI* max maximal left atrial volume index, *LGE* late gadolinium enhancement, *LVEDVI* I Left ventricular end-diastolic volume index, *LVMI* LV mass index

**Table 7:** Stepwise multivariate proportional hazard analyses for combined outcome

Variable	Combined outcome	
	P	HR (95%CI)
Age	< 0.001	1.04 (1.02–1.07)
LVMI	0.007	1.01 (1.00–1.02)
LGE	0.031	1.03 (1.01–1.05)
Total LAEF	0.031	0.97 (0.95–1.00)

In this multivariate model, booster εa was included as covariable, reservoir εs and conduit εe were not included as covariable

*LAEF* left atrial empty fraction, *LGE* late gadolinium enhancement, *LVMI* LV mass index

### Reproducibility of fast LA-LAS

Table 8 presents the results of the reproducibility of fast LA-LAS. Intra- and interobserver reproducibility of fast LA εs and LA εe were excellent (ICC: 0.95−0.96; COV: 7.2%–9.6%). ICC of fast LA εa was 0.87 and 0.88, respectively, and COV of fast LA εa was 9.8% and 10.5%, respectively.

**Table 8:** Intra-observer and inter-observer variability for left atrial strain derived by fast approach

LA fast train	Intra-observer		Inter-observer	
	ICC	COV (%)	ICC	COV (%)
Reservoir εs, %	0.95	6.99	0.95	7.17
Conduit εe, %	0.96	8.23	0.95	9.62
Booster εa, %	0.88	9.76	0.87	10.52

## Discussion

In this study, we report the prognostic value of semi-automated fast LA-LAS using standard cine-CMR images in HCM. First, we found that the mean values of fast LA εs, LA εe, and LA εa were impaired in patients with HCM compared to healthy controls; moreover, the values were more impaired in HCM patients with LA enlargement than in those with normal LA size. Second, fast LA εs, LA εe, and LA εa were found to be significantly associated with LA diameter, corresponding LAEF, and LAVI. Third, fast LA εs and εe were independently associated with adverse clinical outcomes in HCM patients, irrespective of the presence of LGE and presence of LA enlargement.

LA morphology and function are important prognostic parameters in HCM. In 2014, the European Society of Cardiology guidelines proposed that LA size should be considered as a risk factor to calculate the 5-year risk of SCD [1]. A number of studies have reported the relationship between LAV and function and long-term outcomes in patients with HCM [11, 20, 21]. In this study, we found a significant association between LA strain and LA diameter, corresponding LAEF, and LAVI.

Leng et al.demonstrated that LA εs, LA εe, and LA εa were impaired in patients with HCM [13], which is consistent with our results. Further, Kaplan–Meier survival analysis revealed that HCM patients with impaired LA εs, LA εe and LA εa were at an increased risk of adverse clinical outcomes. Vasquez et al. retrospectively studied 94 patients with HCM based on echocardiography and found that low LA reservoir and conduit strain were associated with adverse cardiovascular outcomes of HF, stroke, and death during a follow-up of 5.8 ± 3.3 years; however, they did not find a significant relationship between LA contractile strain and adverse cardiovascular outcomes [7]. Recently, Hinojar et al.conducted a CMR-FT study involving 75 patients with HCM and demonstrated that LA strain was associated with all-cause mortality, cardiovascular death, hospital admission related to HF, and lethal ventricular arrhythmias during a mean follow-up of 3.3 ± 1.2 years [11]. Our findings corroborate with those findings on the prognostic value of LA strain. Univariate and multivariate Cox regression analysis showed that fast LA εs and LA εe were significantly associated with adverse clinical outcomes of cardiovascular death, resuscitated cardiac arrest, SCD aborted by appropriate implantable cardioverter-defibrillator discharge, and hospital admission related to HF in patients with HCM (Table 6, Model 2, Model 3). However, we did not find a significant correlation between εa and adverse clinical outcomes. An increase in LA pressure due to pathophysiological changes mainly affected LA εs and εe in patients with HCM (Table 3). LA εa is the intrinsic function of the LA and mainly determined by LA intrinsic contractility [22]. The lower reproducibility of LA εa might be due to smaller amplitude of the strain values and therefore lower signal-to-noise.

LV LGE and LA size have been significantly correlated with adverse clinical outcomes in patients with HCM [9, 23–25]. Therefore, we performed subgroup survival analyses based on the presence of LV LGE and LA size. HCM patients with impaired εs and εe reached a significantly higher rate of the combined outcome, regardless of the presence of LV LGE. Irrespective of LA size, impaired εs and εe also significantly increased the risk of adverse clinical outcomes. However, the prognostic value of LA εa was not found in HCM patients with negative LGE and normal LA size. Moreover, Previous studies have demonstrated that LAVI min is associated with poor clinical prognoses [20, 26]. This finding is consistent with our results (Table 6, Model 1); however, the multivariate Cox regression model established after adding fast LA-LAS did not include LAVI min. The results of subgroup survival analyses based on LAVI showed that irrespective of whether LAVI max and LAVI min were normal or not, impaired LA εs and LA εe were significantly associated with a higher risk of the combined outcome. These results suggested that fast LA εs and LA εe are stronger prognostic factors than LA size, LAVI min, LAVI max, and the presence of LV LGE. Yang et al.have shown that patients with non-obstructive HCM have LA reservoir and conduit dysfunction before LA enlargement [18]. In our research, the LA-LAS also was impaired in HCM patients with normal LA size, and it can detect early changes of LA function in HCM. Therefore, fast LA εs and LA εe provided added prognostic value over LA size and LAV in HCM.

### Limitations

This study has several limitations. First, this was a single-center study. Second, we did not include LA strain rate and diffuse LV tissue characteristics, such as native T1 and extracellular volume fraction in the multiparametric models. Thus, further studies are warranted to completely understand atrial pathophysiology and its interaction with ventricular tissue characteristics to validate our findings in a larger population of patients with HCM.

## Conclusions

Semi-automated fast LA reservoir and conduit strains are independently associated with adverse clinical outcomes in HCM, irrespective of the presence of LGE and LA enlargement.

## Data Availability

The data will be available from the corresponding author on reasonable request.

## Abbreviations

εs: Reservoir strain
εe: Conduit strain
εa: Active (Booster) strain
AV: Atrioventricular
BMI: Body mass index
BSA: Body surface area
bSSFP: Balanced steady state free precession
CMR: Cardiovascular magnetic resonance
CAD: Coronary artery disease
COV: Coefficients of variation
CI: Confidence interval
ECV: Extracellular volume
FT: Feature tracking
HCM: Hypertrophic cardiomyopathy
HF: Heart failure
HR: Hazard ratio
ICC: Intra-class correlation coefficient
ICD: Implantable cardioverter-defibrillator
LA: Left atrium/left atrial
LA-LAS: Left atrial long-axis strain
LAEF: Left atrial emptying fraction
LAV: Left atrial volume
LAVI: Left atrial volume index
LAVI_min_: Minimum left atrial volume
LAVI_max_: Maximal left atrial volume index
LAVI p-ac: Left atrial volume index prior to atrial contraction
LAVI min: Minimal left atrial volume index
LGE: Late gadolinium enhancement
LV: Left ventricle/left ventricular
LVEDVI: Left ventricular end-diastolic volume index
LVEF: Left ventricular ejection fraction
LVESVI: Left ventricular end-systolic volume index
LVMI: Left ventricular mass index
LVOT: LV outflow tract
RV: Right ventricular
RVEDVI: Right ventricular end-diastolic volume
RVESVI: Right ventricular end-systolic volume index
SCD: Sudden cardiac death
SD: Standard derivation
WT: Wall thickness
